# Intrinsic Foot Muscle Cross‐Sectional Area on MRI in People With and Without Plantar Heel Pain: A Cross‐Sectional Observational Study

**DOI:** 10.1002/jfa2.70183

**Published:** 2026-08-01

**Authors:** John W. A. Osborne, Ana M. Azevedo, Hylton B. Menz, Glen A. Whittaker, Matthew Cotchett, Tom Entwisle, David A. Connell, Shannon E. Munteanu, Karl B. Landorf

**Affiliations:** ^1^ Discipline of Podiatry School of Allied Health, Human Services and Sport La Trobe University Melbourne Victoria Australia; ^2^ Ega Moniz School of Health Sciences Almada Portugal; ^3^ Faculty of Sports Science Chulalongkorn University Bangkok Thailand; ^4^ Center of Excellence in Exercise Physiology for Special Populations Chulalongkorn University Bangkok Thailand; ^5^ Fowler Simmons Radiology Adelaide South Australia Australia; ^6^ Imaging @ Olympic Park AAMI Park Melbourne Victoria Australia

**Keywords:** feet, magnetic resonance imaging, muscles, plantar fasciitis, plantar heel pain

## Abstract

**Introduction:**

This study compared the cross‐sectional areas (CSA) of key intrinsic foot muscles in people with and without plantar heel pain (PHP).

**Methods:**

Seventy‐five adults participated in the study: 50 participants with PHP and 25 age‐, sex‐, and body mass index (BMI)‐matched participants without PHP (control group). Cross‐sectional images were generated using 3.0T magnetic resonance imaging (MRI). CSAs of four intrinsic foot muscles—abductor hallucis, quadratus plantae, flexor digitorum brevis and abductor digiti minimi—were measured. The difference in CSA of each muscle was compared between the groups using independent samples *t*‐tests and Cohen's *d* effect sizes were calculated.

**Results:**

Participants were well matched: mean age 49.1 and 48.9 years, women accounted for 58% and 56%, and BMI 30.6 kg/m^2^ and 30.2 kg/m^2^ in the PHP and control groups, respectively. There were no significant differences in mean CSAs for all four muscles (*p* > 0.05) between the PHP and control groups, and effect sizes were tiny or very small.

**Conclusion:**

This study found no differences in muscle CSAs of key intrinsic muscles of the foot in people with and without PHP, indicating that people with PHP do not have key intrinsic foot muscle size deficits. As such, intrinsic muscle size does not appear to be a logical therapeutic target for PHP. Clinicians translating our findings into practice should, therefore, consider whether strengthening interventions targeting intrinsic muscles in PHP are warranted.

## 1 Introduction

Plantar heel pain (PHP) is a common condition, with up to 10% of the adult population being affected [1, 2]. It can have negative physical and psychological effects including pain on activity and on first steps after rest [3], poor function [4], and poor health‐related quality of life [5, 6]. While several factors such as obesity [7, 8, 9], and prolonged weight bearing [10, 11] have been associated with the development of PHP, reduced muscle strength has also been proposed as an associated factor [8].

If people with PHP have reduced muscle strength and/or size (a proxy for muscle strength) [12] it provides a potential target for muscle strengthening interventions. A previous systematic review by the authors [13] found reduced foot muscle strength and size in people with PHP compared to those without. However, the evidence reviewed was inconsistent with substantial heterogeneity between studies, primarily due to differing measurement methods and results [13]. Furthermore, the Grading of Recommendations Assessment, Development and Evaluation (GRADE) rating for ‘weakness of the lesser toe plantar flexors' associated with PHP was judged to be very low in certainty suggesting that future research may change this finding. Accordingly, whether foot muscle strength or size deficits are a factor in the development of PHP remains unclear.

Measuring foot muscle strength, such as for the toe plantar flexors, is difficult due to anatomical and functional issues, mainly the difficulty isolating intrinsic from extrinsic muscles during digital plantar flexion testing [14]. In turn, muscle size has been considered a valid proxy for muscle strength [12]. While, the association is not certain, some studies have shown a positive correlation between size and strength, particularly for flexor digitorum brevis [12, 15].

Several studies have measured muscle size using medical imaging in people with PHP [16, 17, 18]. Hogan et al. [18] found no difference in abductor hallucis (AbdH) CSA between 16 participants with PHP and 16 age‐, sex‐ and body‐mass matched participants without PHP. They used ultrasound to measure CSA, however Magnetic Resonance Imaging (MRI) provides superior image quality and CSA measurement precision, particularly with higher magnetic field strengths [19, 20, 21, 22]. Using MRI, Chang et al. [16] found a statistically significant reduction in forefoot but not rearfoot muscle volume between the symptomatic and asymptomatic limbs of eight people with unilateral PHP. In contrast, Cheung et al. [17] found 10 runners with PHP had a significant reduction in rearfoot muscle volume compared to 10 age‐, sex‐ and body‐mass matched controls. The studies discussed above used relatively small samples, measured different muscle groups or muscles, and importantly, did not adequately assess reliability of the assessors. In addition, the two MRI studies used relatively low magnetic field strength MRIs at 1.5T, which provides lower quality images and less accurate visualisation of small anatomic structures compared to higher magnetic field strengths, such as 3.0 T MRI [21]. Consequently, these previous studies provide somewhat conflicting findings, there is the possibility that their measurements may not be reliable, and they used imaging that is sub‐optimal by today's standards.

Considering the above, further research is needed with larger samples, assessor reliability testing, and better‐quality MRI imaging. This study aimed to compare the muscle CSAs of four key intrinsic foot muscles in people with and without PHP using 3.0T MRI.

## 2 Methods

Some of the methods outlined below have been reported in earlier publications related to this study [6, 10, 23]. Ethical approval was obtained from the La Trobe University Human Ethics committee application 14‐0001. All participants provided written, informed consent prior to recruitment into the study.

### 2.1 Study Design

This was a cross‐sectional observational study and is reported in accordance with the Strengthening the Reporting of Observational Studies in Epidemiology (STROBE) guidelines [24].

### 2.2 Participants

The participants were 75 community‐dwelling adults of either sex from Victoria, Australia. There were two groups of participants: (i) a group of 50 participants with PHP, and (ii) a control group of 25 participants without PHP (i.e., a ratio of 2:1 of participants with PHP to control participants without PHP). Participants in the control group were matched to PHP group by age (± 5 years), sex, and BMI (± 10%).

#### 2.2.1 Inclusion and Exclusion Criteria

Participants were included in the study if they:

- Were aged 18 years or over;
- Had uni‐ or bilateral PHP for at least 1 month that was confirmed on initial examination (if recruited to the PHP group);
- Were able to speak basic English, so they could provide informed consent prior to participation, follow instructions during the project, and answer questions related to the study accurately.

Participants were excluded from the study if they:

- Had any conditions (e.g., pregnancy, pacemaker, metal fragments, etc.) that would have precluded them from having the medical imaging related to the over‐arching study,
- Had any self‐reported inflammatory arthritis (e.g., seronegative arthropathy), endocrine/neurological condition (e.g., diabetic peripheral neuropathy, stroke, etc.) or surgery (e.g., amputation, joint fusion, etc.) that affected lower limb sensation or their ability to walk/run.

#### 2.2.2 Recruitment

Participants were recruited via several methods including: advertising posters placed at relevant locations (e.g., La Trobe University, private and public health clinics, sporting and senior citizens clubs), the Health Sciences Clinic at La Trobe University, advertisements on relevant websites related to health, direct referral from health care practitioners, acquaintances of the investigators involved with the study, and snowball sampling. Recruitment commenced on 12 January 2015 and was completed on 26 October 2018.

#### 2.2.3 Sample Size

The sample size of 75 was one of convenience and was dependent on an over‐arching study evaluating the differences in medical imaging findings in people with and without PHP. The recruitment ratio of two participants with PHP to one control participant without PHP was decided on to minimise the burden of recruiting age‐, sex‐, and BMI‐matched control participants.

### 2.3 Settings

Participant information and characteristic data were collected in one of three settings: (i) a research room in the Health Sciences Clinic at La Trobe University in Melbourne, Australia, (ii) a health science clinical tutorial room at La Trobe University, or (iii) in a room at the participant's home with a hard floor (e.g., linoleum, concrete or wood). MRIs were performed at Imaging @ Olympic Park, Melbourne, Australia.

### 2.4 Protocol and Data Collection

Data were collected using a standardised assessment form relating to participant characteristics, medical history including prescribed medication, education, occupation, activities, comorbidities, foot problems, pain and function, foot posture, footwear, and health status as part of a previous study [10, 23].

#### 2.4.1 Participant Characteristics

Participants had their height (m) and weight (kg) measured; subsequently, their BMI was calculated in kg/m^2^ [25]. In addition, their waist and hip circumferences were measured (cm); subsequently their waist‐hip ratio was calculated.

To assess the level of pain experienced by PHP participants, a 100 mm visual analogue scale (VAS) was used for measurement [26]. If they were a participant with PHP, they were also asked for the duration of their symptoms.

Participants were asked to report their highest level of education they had completed, which was categorised as follows: (i) no formal, (ii) less than primary school, (iii) primary school completed, (iv) high school (or equivalent) completed, (v) tertiary and further education completed, (vi) college\university completed, (vii) postgraduate degree completed, (viii) don't know, (ix) other (please state).

Participants had their foot posture measured using the Foot Posture Index‐6 (FPI) and Arch Index (AI). The FPI and the AI have been shown to be valid and reliable measures of foot posture [27, 28, 29].

Ankle joint dorsiflexion was measured while the participants were weight bearing using a lunge test, with the knee extended and knee flexed [30, 31]. These tests have been shown to have high to excellent intra‐assessor and inter‐assessor reliability when used by experienced testers [30, 31].

Physical activity (i.e., overall physical activity) was measured by the Stanford Activity Questionnaire (SAQ), which was expressed as kilocalories expended per day [32, 33]. The SAQ has been found to have adequate validity and reliability [32, 34].

More detailed methodological information about the measurements outlined above can be viewed in previous related publications [10, 23].

#### 2.4.2 MRI Protocol

MRI images were generated using a Philips 3.0 T scanner. Images were obtained of the midfoot and rearfoot only, excluding the distal metatarsals and phalanges using an 8‐channel foot/ankle receiver coil. Due to this imaging protocol (i.e., of the over‐arching study), we could not measure the entire muscle volume and were limited to CSA only. CSA is a valid measure for intrinsic foot muscle size and has a moderate correlation to intrinsic foot muscle strength [35]. Participants were positioned with the foot approximately perpendicular to the leg, and each foot was scanned independently. Coronal plane Proton Density Weighted (PDW) images were used for data collection and slice thickness was 1.5 mm. All MRI data were anonymised and exported in DICOM format, converted to NiFTI format, and then exported to 3D imaging software (ITK‐SNAP).

**FIGURE 1:**
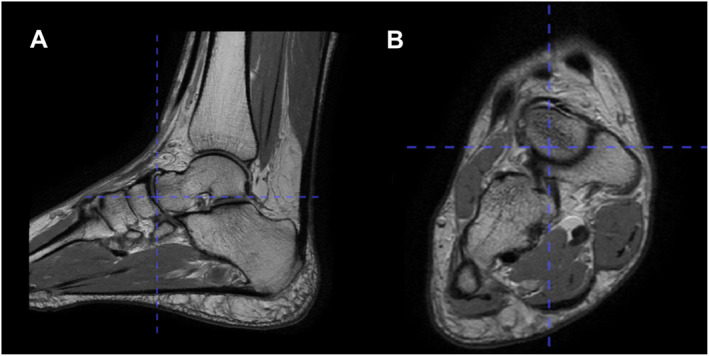
Sagittal and coronal plane MRI representing the location of interest. (A) Sagittal plane image with the blue cross‐hairs indicating the location of interest (talonavicular joint). (B) Coronal plane image with the blue cross‐hairs indicating the centre of the joint of interest. Both images (A) and (B) were aligned and correlated to ensure accuracy of measurement.

Abductor hallucis (AbdH), quadratus plantae (QP), flexor digitorum brevis (FDB), and abductor digiti minimi (AbdDM) were determined as the muscles of interest because of their proximity to the insertion of the plantar fascia and potential association with PHP. Three investigators (JWAO, AMA, and KBL) conducted pilot testing in June 2024, referencing an anatomy textbook [36], ultrasound [37, 38], and MRI studies [19, 39] to determine the location of interest for measurement, which was determined to be the talonavicular joint. The talonavicular joint was chosen because of its ease to consistently locate, while providing a maximised CSA for all muscles measured. For consistency and accuracy, the talonavicular joint was cross‐referenced in both sagittal and coronal planes. If a participant had multiple slices through the location of interest, the centre most sagittal plane slice and most proximal coronal plane slice was used (Figure 1).

#### 2.4.3 Measurement Process

**FIGURE 2:**
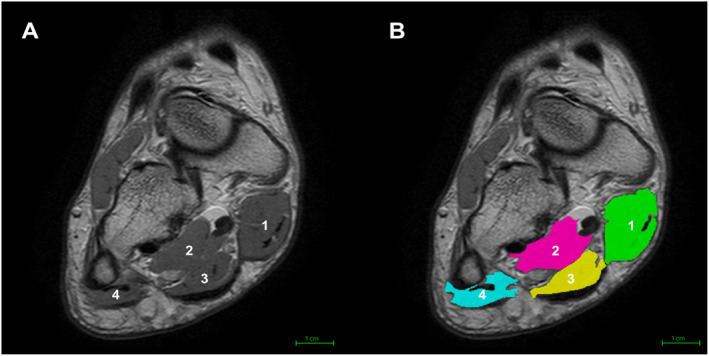
Muscle segmentation examples at the talonavicular joint (both are coronal plane images). Muscles are numbered and shaded as follows; 1 (green) = abductor hallucis, 2 (pink) = quadratus plantae, 3 (yellow) = flexor digitorum brevis and 4 (blue) = abductor digiti minimi. (A) is the coronal plane prior to measuring of area of muscle, (B) shows the area measurement of each muscle as highlighted in respective colours.

Two assessors (JWAO, a registered podiatrist with more than 10 years of experience, and AMA, a registered physiotherapist with 19 years of experience) were blinded to group allocation and measured CSA of each muscle for all 75 participants. The cross‐sectional outline was made in the coronal view using ITK‐SNAP (Version 4.0), which has been used in previous studies for measurement of anatomical structures (Figure 2) [40, 41]. Each muscle was manually traced using a trackpad or mouse, and the segmented area was automatically filled. The outline used the fascial border of each muscle and did not include any fat or other structures located at or on the border of each muscle. Tendon, fat and other structures within the muscle were included as part of the measurement. After each outline was made, the internal segment was filled and the resulting area was measured within ITK‐SNAP. This measurement was exported as a volume calculation of the segmented area. The CSA of each muscle was calculated based on the volume of the segmented area divided by the slice thickness (1.5 mm). The results were exported to Microsoft Excel (Microsoft 365, Microsoft, Redmond, WA, USA).

### 2.5 Statistical Analysis

Data analyses were performed in International Business Machines (IBM) Statistical Package for the Social Sciences (SPSS) Version 29.0 (IBM Corporation). Categorical data were presented as number (%) and associations were tested using Chi‐squared analyses. Nominal data were presented as medians (interquartile ranges) and differences between groups were tested using Mann Whitney *U* tests. All continuous variables for both groups were initially explored for normality prior to inferential analysis. Normally distributed data were presented as means (standard deviations [SDs]) and differences between groups were tested using independent samples *t*‐tests. Any variables found to be not normally distributed were handled non‐parametrically and median (interquartile ranges) were reported and differences between groups were tested using Mann Whitney *U* tests.

Prior to any hypothesis testing being conducted on the muscle CSA data, reliability of the assessment method was undertaken using the two assessors (JWAO and AMA). Both intra‐ and inter‐assessor reliability were assessed on 10 randomly chosen participants (5 from the PHP group and 5 from the control group) using intraclass correlation coefficients (ICC Model 3,1), with the type used being absolute agreement [42]. Once intra‐ and inter‐assessor reliability were determined to be satisfactory, only the most reliable assessors' measurements were used in the final analysis of all 75 participants (i.e., comparing the muscle CSAs in the PHP group to the no PHP group) to avoid artificially decreasing the variability of the data by averaging two sets of measurements.

Following reliability testing and assessments of all 75 participants, differences between the two groups in muscle CSAs were investigated using independent samples *t*‐tests. If Levene's Test for Equality of Variances was statistically significant, results were adjusted as equal variances were not assumed. The critical level of statistical significance was set at *p* < 0.05. Cohen's *d* effect size values were also calculated for the differences in muscle CSAs between the two groups, and interpretations of the effect sizes were taken from Sawilowsky [43], which are < 0.01 = tiny, ≥ 0.01 = very small, ≥ 0.2 = small, ≥ 0.5 = medium, ≥ 0.8 = large, ≥ 1.2 = very large, and ≥ 2.0 = huge.

## 3 Results

There were 50 participants in the PHP group and 25 participants in the control group.

### 3.1 Participant Characteristics

Participants were well matched with no significant differences between the two groups (Table 1). The mean age was 49.1 years in the PHP group and 48.9 years in the control group, with a range for all participants in the study of 23–75 years. Women made up 58% of the PHP group and 56% of the control group. The mean BMI was 30.6 kg/m^2^ in the PHP group and 30.2 kg/m^2^ in the control group, with a range for all participants in the study of 20.1–47.7 kg/m^2^. In addition, measures of central obesity (e.g., waist‐hip ratio) were not significantly different between the two groups, as were foot posture, ankle dorsiflexion measures and activity levels.

**TABLE 1:** Participant characteristics—values are means (SDs) unless otherwise stated.

Variable	PHP group (*n* = 50)	Control group (*n* = 25)	Mean difference (95% CI)	*p*‐value
Age (years)	49.1 (11.6)	48.9 (9.9)	0.2 (−5.2, 5.6)	0.947
Sex—no. of females (%)	29 (58)	14 (56)	N/A	0.869^a^
Height (m)	1.68 (0.10)	1.73 (0.12)	−0.05 (−0.11, 0.00)	0.051
Weight (kg)	86.1 (17.5)	90.3 (21.4)	−4.2 (−13.4, 5.0)	0.370
BMI (kg/m^2^)	30.6 (6.2)	30.2 (7.2)	0.4 (−2.8, 3.6)	0.813
Waist circumference (cm)	100.9 (11.4)	101.7 (19.4)	−0.8 (−9.3, 7.8)^b^	0.858^b^
Hip circumference (cm)	112.0 (12.6)	111.1 (15.3)	0.9 (−5.7, 7.5)	0.784
Waist‐hip ratio	0.90 (0.06)	0.91 (0.09)	−0.01 (−0.05, 0.03)	0.614
Duration of symptoms (months)				
Median (IQR)^c^	6.5 (3.0–12.0)	0.0 (0.0–0.0)^d^	N/A	N/A
Education level—category				
Median (IQR)	6 (4–7)	6 (5–6.5)	N/A	0.785^e^
Foot posture				
Foot posture index	4.3 (2.8)	3.4 (3.0)	−0.9 (−2.3, 0.5)	0.198
Arch index	0.20 (0.10)	0.21 (0.05)	0.01 (−0.02, 0.04)	0.554
Ankle joint dorsiflexion (°)				
Lunge test—knee extended	32.5 (6.4)	35.5 (6.5)	3.0 (−0.1, 6.1)	0.054
Lunge test—knee flexed	40.1 (7.8)	41.9 (7.5)	1.8 (−1.8, 5.5)	0.321
Activity level (kilocalories expended per day)	3745 (1012)	3689 (1034)	56 (−441, 554)	0.823

Abbreviations: CI, confidence interval; cm, centimetre; IQR, interquartile range; kg, kilogram; m, metre; N/A, Not applicable; PHP, plantar heel pain; SD, standard deviation.

^a^ *p*‐value relates to Chi‐squared test.

^b^ Mean difference, 95% CIs and *p*‐value adjusted as Levene's Test for Equality of Variances was significant (*p* < 0.05).

^c^ Median (IQR) reported as variable not normally distributed.

^d^ control group did not have PHP.

^e^ *p*‐value relates to Mann‐Whitney *U* test.

Regarding symptoms in the PHP group, the mean (SD) first‐step pain was 53.3 (26.5) mm, pain on the day of their assessment was 39.7 (20.7) mm, and pain in the last 7 days was 50.8 (24.5) mm. The median (IQR) duration of symptoms was 6.5 (3.0–12.0) months in the PHP group with a total range of 1.0–80.0 months.

### 3.2 Reliability Testing

Prior to comparing muscle CSA between participants in the PHP and control groups, reliability testing (absolute agreement) was undertaken. Both intra‐ and inter‐tester reliability for the four muscles were excellent with ICCs ranging from 0.97 to 1.00 and 0.92 to 0.99, respectively (Table 2). Accordingly, it was determined that the assessment method was reliable.

**TABLE 2:** Results for intra‐ and inter‐assessor reliability (absolute agreement)—values are ICCs (95% CIs).

Muscle	A1 T1 versus A2 T1	A1 T2 versus A2 T2	A1 T1 versus A1 T2	A2 T1 versus A2 T2
AH	0.985 (0.886, 0.997)	0.987 (0.847, 0.997)	1.000 (0.999, 1.000)	0.998 (0.990, 0.999)
QP	0.922 (0.688, 0.981)	0.955 (0.829, 0.989)	0.997 (0.987, 0.999)	0.979 (0.920, 0.995)
FDB	0.958 (0.842, 0.989)	0.961 (0.858, 0.990)	0.999 (0.997, 1.000)	0.967 (0.875, 0.992)
ADM	0.962 (0.862, 0.990)	0.965 (0.869, 0.991)	1.000 (0.998, 1.000)	0.998 (0.994, 1.000)

*Note:* 1. The ICC model used was ICC (3,1), which is a two‐way mixed, single measure.

Abbreviations: A1, assessor 1; A2, assessor 2; ADM, abductor digiti minimi; AH, abductor hallucis; CI, confidence interval; FDB, flexor digitorum brevis; ICC, intraclass correlation coefficient; QP, quadratus plantae; T1, assessment time 1; T2, assessment time 2.

### 3.3 Comparison of the Muscle Cross‐Sectional Areas

There were no significant differences in muscle CSAs for all four muscles assessed between the PHP and the control groups (Table 3). All effect sizes were interpreted as tiny or very small.

**TABLE 3:** Comparison of the muscle cross‐sectional areas in mm^2^ for the PHP group (*n* = 50) and the control group (*n* = 25).

Muscle	PHP group: Mean (SD)	Control group: Mean (SD)	Mean difference (95% CI)	*p*‐value	Effect size^a^ (95% CI)
					Interpretation^b^
AH	573.0 (183.7)	571.5 (129.7)	1.5 (−80.5, 83.4)	0.971	0.009 (−0.471, 0.489)
					Tiny
QP	459.6 (124.9)	453.2 (186.6)	6.4 (−77.4, 90.2)^c^	0.878	0.043 (−0.437, 0.523)
					Very small
FDB	548.6 (151.5)	530.0 (131.3)	18.5 (−52.3, 89.4)	0.604	0.128 (−0.353, 0.608)
					Very small
ADM	325.7 (130.1)	305.8 (149.5)	19.9 (−46.9, 86.7)	0.554	0.146 (−0.336, 0.626)
					Very small

Abbreviations: ADM, abductor digiti minimi; AH, abductor hallucis; CI, confidence interval; FDB, flexor digitorum brevis; PHP, plantar heel pain; QP, quadratus plantae; SD, standard deviation.

^a^ Cohen's *d* effect size.

^b^ Interpretations for effect sizes were taken from Sawilowsky [43].

^c^ Levene's Test for equality of variances was statistically significant, so results adjusted as equal variances not assumed.

## 4 Discussion

The aim of this study was to compare the CSAs of four key intrinsic foot muscles in people with and without PHP. Our findings demonstrate that there was no significant difference in muscle CSA for those with PHP compared to those without (all *p* > 0.05). Further, effect sizes were tiny or very small, reinforcing that any differences in CSAs can be interpreted as not being important. Our study included a substantially larger number of participants than previous studies and used 3.0T MRI, which provided clearer delineation of the foot muscles for measurement compared to previous research. It also assessed both intra‐ and inter‐assessor reliability of the assessors involved in the study. Thus, our study is an improvement on previous studies.

Our findings are similar to two previous studies that found no difference in intrinsic muscle size [16, 18], and one study that found no difference in extrinsic muscle CSA [44] between people with and without PHP. However, the first of these studies by Chang et al. [16] assessed different variables to our study; among other variables, the entire rearfoot muscle volume was measured using 1.5T MRI images. Even though we did not combine all of the rearfoot muscles like Chang et al. [16], nor did we measure muscle volume, each of the four individual muscles we measured (including AbdH) were not significantly different in their CSA. The second study by Hogan et al. [18] measured CSA, similar to us, however they used ultrasound as opposed to our study that used 3.0T MRI. They only measured one muscle, AbdH, finding no significant difference in CSA between participants with and without PHP. Regarding the study that assessed extrinsic muscle size [45], the findings from this study were similar to ours in that the thickness and CSA of all measured muscles (tibialis posterior, gastrocnemius medialis and soleus) did not show significant differences between people with and without plantar heel pain when measured with ultrasound. In contrast, our findings are different to Cheung et al. [17] who used 1.5T MRI images. They found that there was a difference in normalised rearfoot muscle volume between runners with and without PHP. The results showed those with PHP had 195.7 mm^3^/kg lower muscle volume than those without [17]. Again, caution should be made with this comparison as our study only measured muscle CSA and not volume.

Regarding the clinical implications of our study, intrinsic muscle size does not appear to be a logical therapeutic target for PHP. Clinicians translating our findings into practice should, therefore, consider whether strengthening interventions targeting intrinsic muscles in PHP are warranted. Instead, clinicians are likely better directed to address other factors associated with PHP first, such as obesity [7, 8, 9], time standing on hard surfaces [8, 10, 11], and footwear [8, 10], which have more robust evidence that they contribute to the development of the condition. However, we cannot discount the use of strengthening exercises in the management of PHP as they may have effects beyond muscle hypertrophy alone.

It should also be noted that other researchers have found systemic impacts of foot pain, which suggests interventions should address more global health rather than isolated muscle morphology [46, 47]. In addition, PHP is likely not isolated to plantar fascia pathology [48], so focussing on muscle size or strength and its relationship to the plantar fascia alone may not lead to the best outcomes. e.g., studies have found that people with PHP may also have changes in the plantar fat pad [49, 50] and bone marrow oedema of the calcaneus [51, 52]. Accordingly, PHP is a condition that affects multiple anatomical structures, so interventions should be broad and target key associated factors in its pathogenesis such as reducing weight, minimising weight bearing on hard surfaces, and restricting use of hard‐soled shoes.

Several limitations of our study should be considered. First, this study was part of an over‐arching study on imaging for PHP, so the MRI images only included muscles at the rear of the foot. This limitation meant we could not measure the entire muscle volume and were limited to CSA only. However, CSA is a valid measure for intrinsic foot muscle size and has a moderate correlation to intrinsic foot muscle strength [35]. Second, the sample, while reflective of the general population with PHP may not reflect sub‐populations (i.e., the athletic population with PHP). Third, we included fascia, tendon, and fat in our cross‐sectional muscle measurements if those tissues were within the boundaries of each muscle. This was done to simplify data collection as it was too difficult to segment multiple structures within a muscle, so we may have overestimated the CSA of the muscles we measured. Nevertheless, we used this measurement technique for both groups, so our technique was consistent for comparisons between the two groups. Fourth, we measured muscle size and not muscle strength or quality, so we cannot make conclusions regarding those parameters. So, although we make inferences that muscle size is associated with muscle strength and quality, we cannot be certain of this; although, some studies have shown a positive correlation between both size and strength. Future studies could consider the relationship between foot muscle size and strength in those with PHP and healthy populations, and whether foot muscle strengthening has effects beyond size changes alone in people with PHP.

## 5 Conclusion

In summary, the results of this study show that there is no difference in the CSA of key intrinsic foot muscles in those with and without PHP. Our study had a large and well‐matched sample of participants and used higher quality images than previous studies on this topic. The findings suggest that intrinsic foot muscle size deficits are likely not a significant factor in PHP. Accordingly, clinicians should consider focussing their attention on established associated factors such as obesity, time standing on hard surfaces, and footwear.

## Author Contributions

**John W. A. Osborne:** conceptualisation, methodology, formal analysis, writing – original draft, writing – review and editing. **Ana M. Azevedo:** conceptualisation, methodology, formal analysis, writing – review and editing. **Hylton B. Menz:** conceptualisation, methodology, formal analysis, writing original draft, writing – review and editing. **Glen A. Whittaker:** conceptualisation, methodology, writing – original draft, writing – review and editing. **Matthew Cotchett:** writing – original draft, writing – review and editing. **Tom Entwisle:** conceptualisation, methodology. **David A. Connell:** conceptualisation, methodology. **Shannon E. Munteanu:** conceptualisation, methodology, writing – review and editing. **Karl B. Landorf:** conceptualisation, methodology, formal analysis, writing – original draft, writing – review and editing, supervision.

## Funding

This project was funded from research grants provided by the Sport, Exercise & Rehabilitation Research Focus Area and the School of Allied Health at La Trobe University.

## Ethics Statement

Ethical approval was sought from the La Trobe University Ethics committee application No. 14‐0001.

## Consent

All participants provided written, informed consent prior to recruitment into the study.

## Conflicts of Interest

Hylton B Menz and Karl B Landorf are Emeritus Editors of the Journal of Foot and Ankle Research. It is a journal policy that editors are removed from the peer review and editorial decision‐making processes for manuscripts they have co‐authored.

## Data Availability

Data are available upon reasonable request. Data that support the findings of this study can be requested from both the corresponding author (John W A Osborne) and the senior author (Karl B Landorf) via their respective emails (j.osborne@latrobe.edu.au and k.landorf@latrobe.edu.au).
